# DNA barcode reference library for the West Sahara-Sahel reptiles

**DOI:** 10.1038/s41597-022-01582-1

**Published:** 2022-08-01

**Authors:** Guillermo Velo-Antón, Margarida Henrique, André Vicente Liz, Fernando Martínez-Freiría, Juan Manuel Pleguezuelos, Philippe Geniez, Pierre-André Crochet, José Carlos Brito

**Affiliations:** 1 Universidade de Vigo, Departamento de Ecoloxía e Bioloxía Animal, Grupo GEA, Vigo, E-36310 Spain; 2 CIBIO, Centro de Investigação em Biodiversidade e Recursos Genéticos, InBIO Laboratório Associado, Campus de Vairão, Universidade do Porto, 4485-661 Vairão, Portugal; 3 BIOPOLIS Program in Genomics, Biodiversity and Land Planning, CIBIO, Campus de Vairão, 4485-661 Vairão, Portugal; 4 Departamento de Biologia, Faculdade de Ciências, Universidade do Porto, 4099-002 Porto, Portugal; 5 Departamento de Zoología, Facultad de Ciencias, Universidad de Granada, E-18071 Granada, Spain; 6 CEFE, EPHE-PSL, CNRS, Univ Montpellier, Biogéographie et Ecologie des Vertébrés, Montpellier, France; 7 CEFE, CNRS, Univ Montpellier, EPHE, IRD, Montpellier, France

**Keywords:** Herpetology, Biodiversity

## Abstract

DNA barcode reference libraries are now continuously produced for the tree of life, which are essential pillars for the study of biological diversity. Yet, our knowledge about global diversity is largely limited in undersampled regions such as the largest warm desert, the Sahara-Sahel. This dataset provides a DNA barcode reference library for the reptiles of the Western Sahara-Sahel (WSS) and neighbouring countries across this region. It includes 760 barcodes from 133 reptile taxa, distributed in 23 families, and covering the intraspecific diversity of some species. A total of 84 species were collected in the WSS (83% of the total reptile species richness) over 18 overland field expeditions conducted since 2003. DNA barcodes resulted in a high success rate (95%) of species identification and barcoding gap analysis highlighted the effectiveness of the COI fragment as a barcode marker for the WSS reptiles. This dataset represents a comprehensive and reliable DNA reference library for the WSS, filling an important biodiversity gap across a remote and hard-to-sample region.

## Abstract (summary)

**Table:** 

Measurement(s)	reptile diversity
Technology Type(s)	DNA barcoding
Factor Type(s)	DNA barcode sequencing
Sample Characteristic - Organism	reptiles
Sample Characteristic - Environment	terrestrial
Sample Characteristic - Location	Western Sahara-Sahel

## Background & Summary

Global biodiversity is currently undergoing an unprecedented crisis^1^ caused by the devastating effects of human activities on wildlife^2^. Yet, the knowledge available about overall global biodiversity is very limited^3^ because many species have not been formally described, and certain geographic regions are still undersampled, which results in underestimation of biodiversity loss^4^. The Sahara Desert, together with the neighbouring arid Sahel, is the largest warm desert in the world and both represent two major ecoregions of the African continent, covering about 11,230,000 km^2^ ^5^. Molecular taxonomy studies developed over the last decade within the Sahara-Sahel highlighted the need of urgent research to identify the hidden vertebrate diversity present in this arid and remote region^6,7^.

**Fig. 1:**
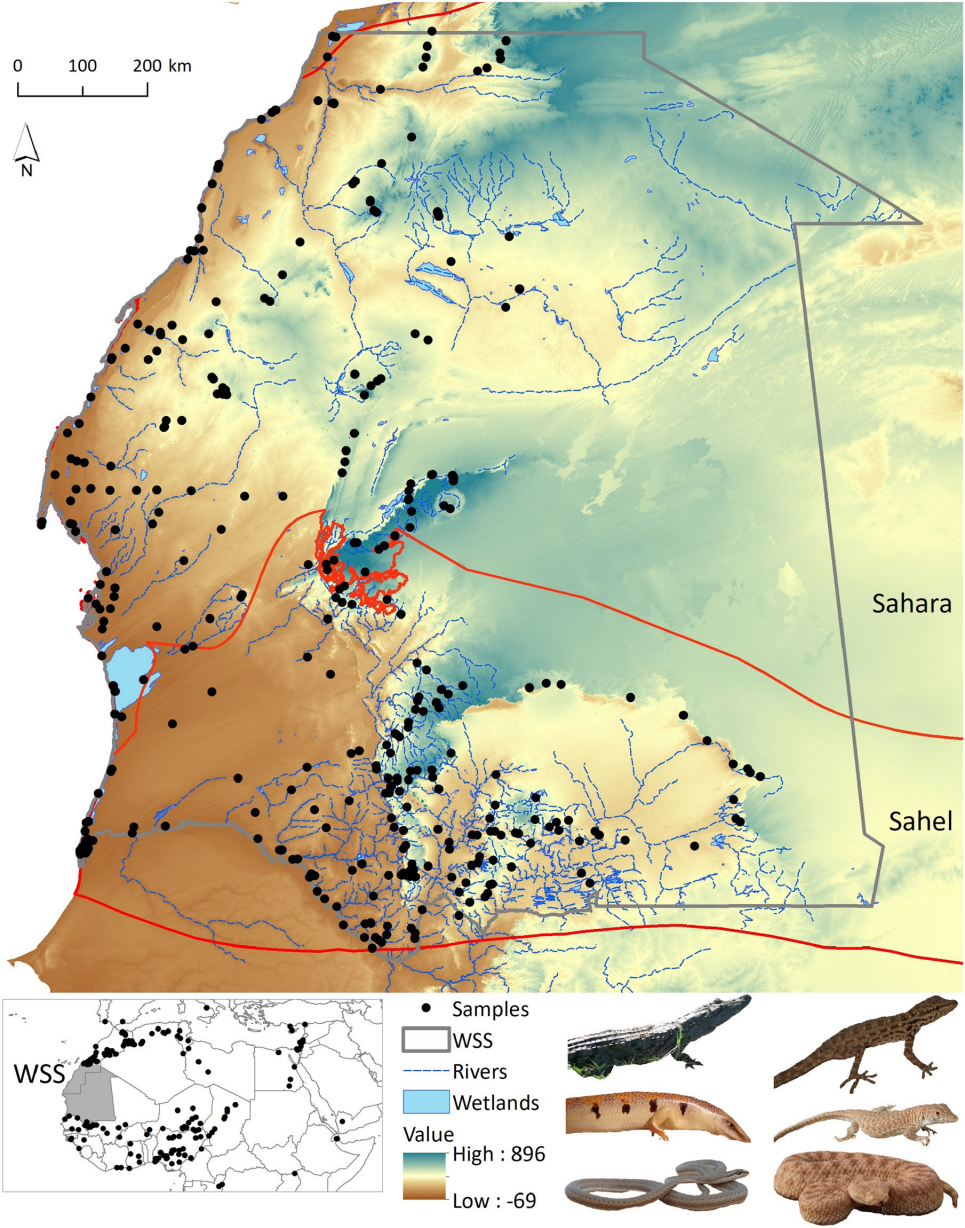
Distribution of samples for the WSS reptile dataset showing the altitude across this region. The inset below indicates the distribution of samples in neighbouring countries across the Sahara and Sahel ecoregions (delimited by red lines).

The West Sahara-Sahel (hereafter WSS) spreads across Mauritania and southern Morocco and represents a transition zone between the Palaearctic and Afro-tropic biogeographical realms^5^ (Fig. 1), acting as a biogeographic crossroad^6^. A total of 103 reptile species have been identified in the WSS (Species list^8^; IUCN Red List, The Reptile Database), with representatives from several ecoregions (e.g. Mediterranean dry woodlands, Sahara Desert, Sahelian savannahs), making it one of the richest vertebrate groups in this region. Recent molecular studies have detected cryptic diversity in several reptile groups^9–17^, suggesting that a significant amount of diversity remains undescribed. This is mainly due to the extensive information gaps regarding local species richness and individual species across this remote and hard-to-sample area^6^, and is particularly important in the local biodiversity hotspots occurring in the mountains scattered across this region, which has been identified as priority for the conservation of Sahara-Sahel biodiversity^18,19^.

An overall genetic assessment of the reptile diversity occurring in the WSS will help to: i) identify potential cryptic diversity; ii) study evolutionary and landscape processes associated with biodiversity distribution; and iii) contribute to the conservation planning of regional reptile diversity^20^. For this purpose, DNA barcoding comes as a fast and cost-efficient method that uses a single, short, standardized, and highly variable genetic marker for species identification and discovery in groups where mitochondrial DNA (mtDNA) is species-specific^21^. The Consortium for the Barcode of Life (CBOL; http://www.ibol.org/phase1/cbol/) aims at retrieving a DNA barcode for every species on Earth and lead to the development of numerous barcoding initiatives, including the ColdCode that aims at barcoding all herpetofauna^22^. This initiative established the widespread use of cytochrome c oxidase 1 (COI) mitochondrial marker for barcoding studies, resulting in the discovery and assessments of herptile diversity across the globe^20,23^, including the amphibians of the WSS^7^. DNA barcoding can thus aid to bridge the knowledge gap in poorly studied areas by providing means to understand local species diversity^24^. Rapid assessments of biodiversity are extremely important in current conservation decision-making^25^.

This work represents the first DNA barcoding study of the reptiles in the WSS, which aims to: 1) establish a COI reference barcode library for the WSS reptiles; 2) assess the effectiveness of the barcode library for specimen identification using distance-based methods; and 3) identify possible candidate cryptic reptile species.

## Methods

### Study area

The WSS (1,024,538 km^2^) includes nine terrestrial ecoregions^5^ with scattered scarps-like mountains separating sandstone plateaus (Fig. 1). There is a cool, dry season from November to February and a hot, dry season from March to June. Rain falls in a single wet season from December to March in the Sahara part, and from July to September in the Sahel part, and there is a marked north-south gradient in increasing annual precipitation. Dunes, gravel and sand floodplains, compact soil, bare rock and rocky soil, grasslands, and other land-cover types cover most of WSS^26^. The area is intrinsically remote and occasionally affected by regional conflict derived from political instability^6,27^, which hampers regular field surveys.

### Sampling strategy and collection and identification of specimens

**Fig. 2:**
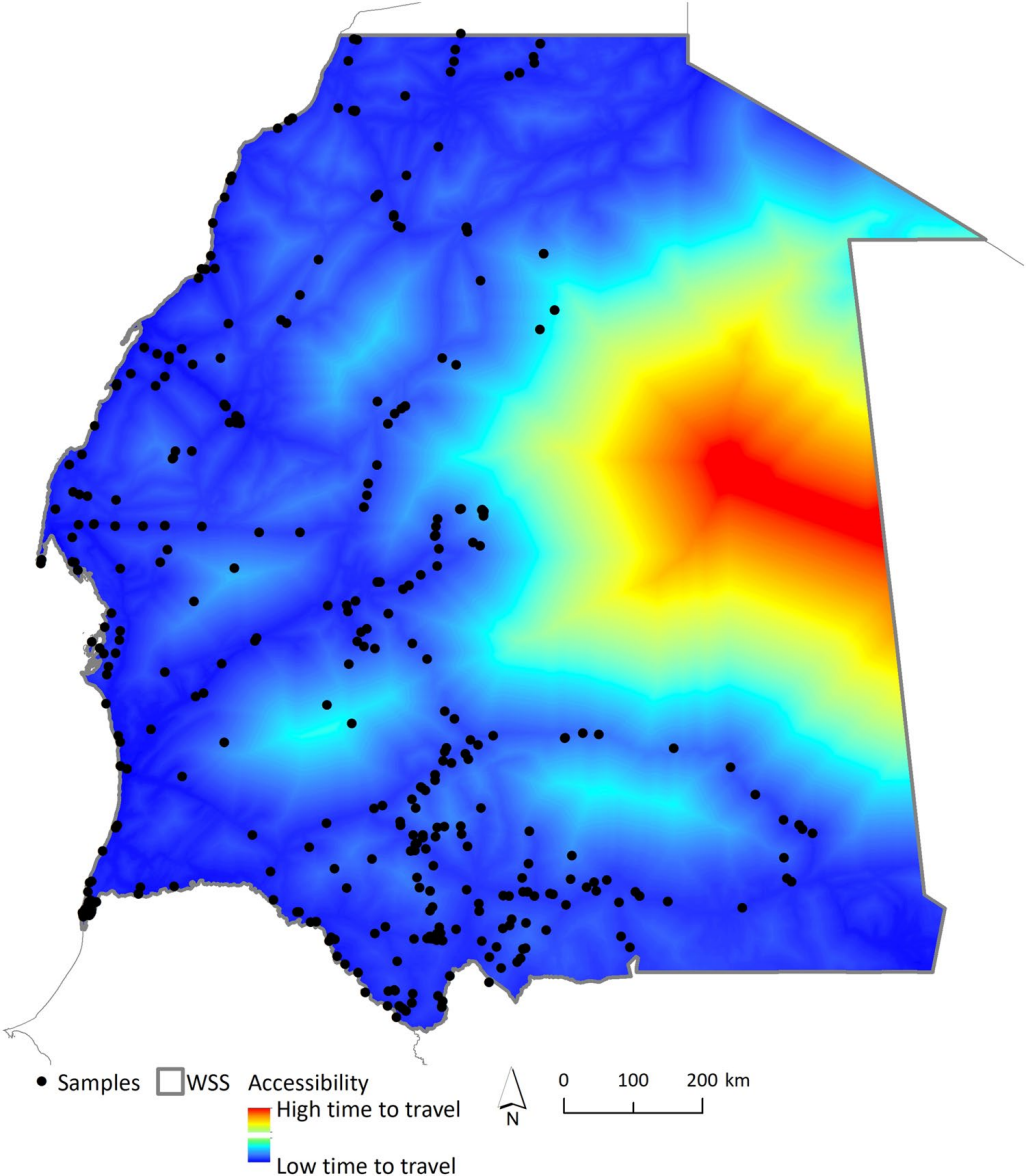
Distribution of samples and accessibility to the study area. Accessibility measured as time to travel to a specific pixel (for details see Weiss *et al*.^28^).

A total of 18 overland field expeditions to WSS were conducted between 2003 and 2020 to collect samples. Field missions were developed annually, from September to December except in 2009 and 2017 (March-May) and 2015 (August). Given the remoteness of the study area and the danger of travelling in some regions (landmines from previous conflicts; Fig. 2), sampling did not follow a stratified approach but was driven by accessibility, being restricted to main routes and tracks^28^ (Fig. 2). Visual encounter surveys in specific habitats and ad-hoc sampling (e.g. roadkill specimens) were used to find reptiles that were collected by hand or pole-noosing. For each specimen, we: 1) collected a tissue sample from tail tip and stored it in 96% ethanol (as well as non-invasive samples, e.g. skin sheds, bones); 2) took reference digital photographs; 3) recorded the spatial location with a GPS (in WGS84 datum); and 4) preliminarily identified it to species level based on external characters following identification keys^29–32^. After data collection, live specimens were returned to their locations. In addition, samples from WSS specimens deposited in the museum collections of MNHN Paris, BEV/CEFE Montpellier, and MHNC-UP Porto, were also included in this dataset, as well as other available samples of studied WSS taxa collected across the Sahara and Sahel ecoregions (Sample list^8^). GenBank sequences from WSS taxa, within (N = 3) or outside the study area (N = 124; 28 sequences with unknown spatial reference), and closely related species were also retrieved to aid barcoding gap analysis, which rely on the difference between maximum intraspecific and minimum interspecific genetic distances.

### DNA barcode sequencing

Total genomic DNA was extracted using the QIAGEN EasySpin Kit or the QIAGEN QIAmp® DNA MicroKit for the samples for which the amount of tissue was limited. For samples where we expected DNA of low quality (tissue collected from dead animals or shed skin), the QIAGEN DNeasy Blood &Tissue Kit was used following manufacturer’s instructions. DNA from museum samples was extracted following an optimized protocol^33^. DNA extractions and subsequent procedures (PCR) were performed in sterile and isolated rooms under special conditions optimized for the manipulation of low-quality DNA.

We used the cytochrome c oxidase 1 (COI) mitochondrial marker, which is the standard barcoding marker for animals (BOLD, http://www.boldsystems.org). A COI fragment was amplified using degenerate primers RepCOI-F (primer forward, 5′-TNTTMTCAACNAACCACAAAGA-3′) and RepCOI-R (primer reverse, 5′ ACTTCTGGRTGKCCAAARAATCA-3′)^34^, except for samples of *Acanthodactylus scutellatus, Pristurus adrarensis* and *Philochortus zolii*, and some samples of *Acanthodactylus longipes*, *Acanthodactylus taghitensis* and *Chalcides delislei* where COI was amplified with the universal primers LCO1490 (primer forward, 5′-GGTCAACAAATCATAAAGATATTGG-3′) and HC02198 (primer reverse, 5′-TAAACTTCAGGGTGACCAAAAAATCA-3′)^35^. A touchdown PCR was performed with the following conditions: initial denaturation at 95 °C for 10 min, followed by an initial phase of 9 cycles of 40 s of denaturation at 95 °C, 30 s of annealing at 52 °C with a decrease in the annealing temperature by 0.5 °C per cycle until the 48 °C, and extension at 72 °C for 45 s, and then followed by a second phase with 31 cycles of 40 s of denaturation at 95 °C, 30 s of annealing at 48 °C, and elongation during 45 s at 72 °C, and a final extension cycle of 10 min at 72 °C. Adjustments of the temperature gradients were done for some species. Quality of PCR products were checked by visual examination in electrophoresis using 2% agarose gel. PCR products were outsourced for Sanger sequencing to Beckman Coulter Genomics (Essex, UK). The sequence chromatograms were visually inspected, assembled, and edited using Geneious Pro v.4.8.5 (Biomatters Ltd.). Sequences were aligned using the MUSCLE version implemented in Geneious Pro v.4.8.5 (Biomatters Ltd.) under default settings^36^. All sequences were translated into amino acids to aid the alignment, and were checked for stop codons to detect the presence of nuclear DNA pseudogenes (NUMTs). Once the absence of NUMTs were confirmed, the sequences were trimmed to the same length.

## Data Records

This reference library contains the following information: 1) Specimen ID; 2) Species ID; 3) Georeferenced data (latitude and longitude in decimal degrees) associated to each specimen collected in the field; 4) sampling date); 5) DNA barcode of each specimen; 6) Institution storing vouchers or tissue/DNA for each specimen; and 7) Photographs of live specimens collected in the field. A specimen was considered as reference for subsequent analysis and interpretation of results when the same identification was obtained from both molecular and morphological assessments. It also contains notes on the taxonomic ID for those specimens in which molecular and morphological identifications disagreed. All data associated with this study is hosted at Figshare^8^. DNA barcodes are available in GenBank (accession numbers ON943478-ON944026) and in BOLD (Ref: REWSS).

A total of 760 barcode sequences from 133 reptile taxa distributed in 23 families were analysed (Sample list^8^). These included: 1) 419 samples from 63 species known to occur in the WSS, collected within the study area; 2) 285 samples of WSS taxa collected outside the study area or lacking spatial reference; and 3) 56 samples from 46 outgroups (i.e. sister/close neighbours of WSS taxa). Samples were selected for analyses in order to cover the described taxonomic diversity and known geographic distribution of each taxon within the WSS, and represent 83% of the total reptile species richness described in the region^29–32^ (Sample list^8^). A total of 472 specimens were identified morphologically to the species level based on external diagnostic characters, from which 376 specimens were sampled in the WSS (Sample list^8^). Specimens where identification to species was not possible (documentation too poor or species pairs too difficult to identify from photos) were not treated as reference samples.

## Technical Validation

We conducted independent morphological identifications by two of the authors (P.A. Crochet and P. Geniez) without regarding the genetic identification nor the geographic origin of the sample. Then, after listing the disagreements in identifications, the sequences and the voucher specimens or pictures were revised a second time to search for possible mistakes in the original identifications. Obvious mistakes in identification or in curation (mixing of photos for example) were corrected, in all other cases the mismatch between genetic and morphological identification was recorded as such.

**Fig. 3:**
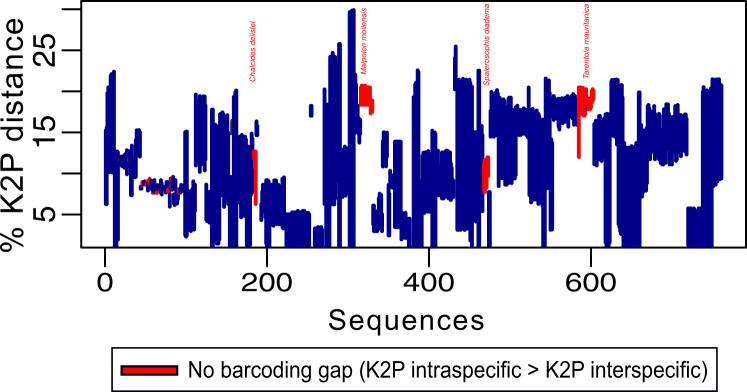
Representation of the barcoding gap for the WSS reptile dataset. Each individual in the dataset is represented by a vertical line, with the top of the line representing the minimum interspecific distance and the bottom of the line representing the maximum intraspecific distance. Barcoding gaps are present if the maximum intraspecific distance is lower than the minimum interspecific distance. Individual lines are color-coded based on the absence (red) or presence (blue) of a barcoding gap. Species for which there is no barcoding gap in all sampled individuals are denoted in red.

To assess the robustness of our library and detect potential cryptic diversity in our dataset (excluding outgroups), we first evaluated the existence of a barcoding gap. A barcoding gap exists when the maximum intraspecific distance of each species is lower than its minimum distance to the nearest neighbour, and thus allows to evaluate the performance of the COI marker as a barcode, but also unveil cryptic diversity. A pairwise distance matrix was first calculated between sequences using the Kimura 2-parameter model (K2P)^37^ to estimate the largest intraspecific distance and the smallest interspecific distance using the statistics *maxInDist* and *nonConDist*, respectively, which are implemented in the R package *spider* v.1.3^38^. We found the presence of a barcoding gap in 92% of the samples, with a lack of barcoding gap in *Chalcides delislei*, *Malpolon moilensis*, *Spalerosophis diadema* and *Tarentola mauritanica*. We also identified a lack of barcoding gap in samples of *Acanthodactylus longipes Agama agama, Dasypeltis scabra* and *Spalerosophis diadema* (Fig. 3; Barcoding gap^8^). Intraspecific genetic distances ranged from 0% to 20.7% (*Malpolon moilensis* showing the highest intraspecific distance). A 2.1% minimum interspecific divergence was found between *Crocodylus suchus* and *C. niloticus*.

**Fig. 4:**
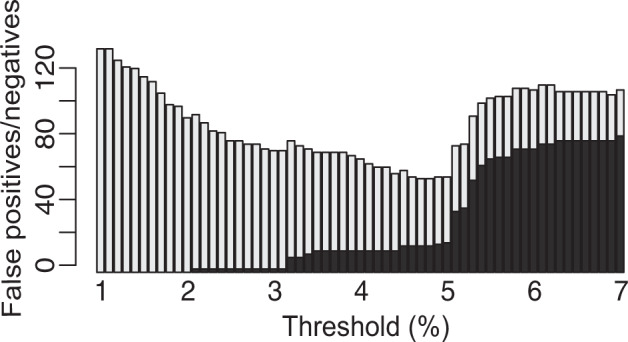
Histogram illustrating the false positive (light grey) and false negative (dark grey) rate of identification of reptiles as pre-set thresholds change.

**Fig. 5:**
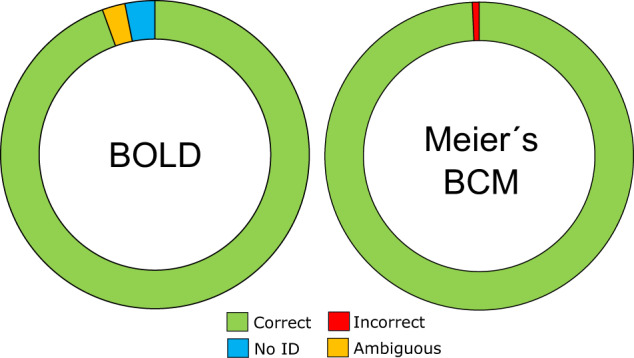
Results from barcoding efficiency methods (BOLD and Meier’s BCM) to determine the consistency of DNA barcodes with currently accepted taxonomy for the WSS reptile dataset.

We also tested for barcoding efficiency (i.e. assignment of barcodes to the species level) in our dataset (excluding outgroups) using two query identification analyses based on genetic distance thresholds. We used the BOLD and Meier’s best close match functions (Meier’s BCM^39^) as implemented in *spider*. These two methods use a threshold based criterion that compares all specimens within the threshold of the query, and then assigns a diagnosis to each identification query: i) “correct match” (within the threshold of the query all matches are the same species); ii) “incorrect match” (i.e. closest match is a different species of the query); iii) “ambiguous match” (i.e. both correct and incorrect species matches within the threshold), and (iv) “no identification” (i.e. no species is identified within the given threshold). We explored a range of threshold values (1%–7%) before choosing the threshold value that minimized the cumulative error (false negative + false positive). Preliminary analyses indicated 5% as the most suited threshold for specimen identification (Fig. 4), which we applied to both methods. We removed species (N = 47) represented by only one sample (singletons) from these analyses and the outgroup samples for this count. BOLD identified 393 (94.5%), 10 (2.4%) and 13 (3.1%) samples from WSS taxa as correct, ambiguous or not identified, respectively. Meier’s BCM identified 414 (99.5%) and 2 (0.5%) WSS samples as correct and incorrect, respectively (Fig. 5). Samples identified as incorrect correspond to single samples of *Chalcides delislei* and *Tarentola mauritanica* from WSS, and *Spalerosophis diadema* and *Dasypeltis scabra* from outside WSS (BOLD and Meier’s BCM^8^).

## Usage Notes

We provide a comprehensive and publicly available DNA barcode library for the West Sahara-Sahel reptile taxa that will allow barcoding or metabarcoding surveys for specimen identification, as well as for biogeographic and evolutionary studies encompassing this region.

Overall, this work improved the current knowledge on species presence, range distribution and levels of genetic structure for WSS reptile fauna. Several results can be highlighted:

A barcoding gap was largely present in our dataset, highlighting the effectiveness of the COI fragment as a barcode marker.

The lack of barcoding gap in a handful of species and the high levels of intraspecific diversity unveiled in a high number of taxa pinpoints the urgent need of further studies and taxonomic re-evaluation of some groups across the WSS, as it has been recently done for other reptiles (e.g. *Mesalina*^40^). Our results suggest potential cryptic diversity at least in the following taxa (showing > 10% of intraspecific divergence): *Acanthodactylus boskianus, Acanthodactylus longipes, Acanthodactylus taghitensis, Agama boulengeri, Chalcides delislei, Chalcides ocellatus*, *Latastia longicaudata*, *Lytorhynchus diadema, Malpolon moilensis*, *Mesalina guttulata, Ptyodactylus oudrii*, *Spalerosophis diadema, Stenodactylus mauritanicus*, *Stenodactylus sthenodactylus, Tarentola chazaliae, Tarentola ephippiata*, *Tarentola mauritanica, Tarentola parvicarinata, Trachylepis perrotetii* and *Tropiocolotes tripolitanus*.

This COI database also contains own and retrieved sequences from GenBank from WSS reptile taxa outside the study region, as well as phylogenetically close neighbours of WSS reptiles. Thus, this reference library is also expected to benefit a large community of researchers studying reptiles across remote and hard-to-sample areas in the vast Sahara-Sahel region.

The main issue with mtDNA barcoding as a tool for specimen identification is the possibility that mtDNA is not species-specific, due to either lack of complete lineage sorting or mtDNA gene flow (introgression) after speciation. Very few barcoding studies explicitly examine this issue by careful identification of specimens independently of genetic results. Here, we evaluated the diagnostic power of COI barcodes by comparing morphological and barcode identification in most specimens (labelled “reference” in Sample list^8^). We confirmed that COI is a reliable tool for specimen identification in most species of reptiles in our dataset.

In a few species pairs (e.g. *Stenodactylus sthenodactylus* and *S. mauritanicus*) or species complexes (e.g. *Acanthodactylus scutellatus* complex), reliable morphological identification from photographs is not possible in all specimens and discordance are more likely to result from morphological misidentifications than mtDNA lineage sharing. Indeed, a recent study combining mtDNA and nuclear data found no instance of mtDNA lineage sharing between species in the *A. scutellatus* complex.

A few (probably) real discordances between morphological and molecular identification remain after discarding possible morphological misidentifications. In one case, such discordance suggests mitochondrial introgression events between sister taxa across contact zones: *Uromastyx nigriventris* and *U. dispar* were found to be non-monophyletic in COI barcodes and although morphological identification of these two species is challenging, the sample suggesting mismatch had a seemingly typical morphology and comes from the contact zone between the two species so the lineage sharing is probably genuine (but would need to be confirmed with nuclear markers). In other cases, the discordances involve taxa where species-level systematics and species limits remain poorly understood and our results are difficult to interpret: *Trapelus boehmei* and the extralimital *T. mutabilis* or the *Tarentola ephippiata* complex (where the subspecies *hoggarensis* is most likely a valid biological species).

Last, nomenclatural instability is another source of confusion when using barcodes for specimen identification. Our samples of *Agama agama* would match samples of *Agama picticauda* in Genbank because of the confused nomenclatural situation in the *Agama agama* complex. We totally agree with the interpretations of a molecular study^41^ that considers the neotype designation for *Agama agama*^42^ as invalid, and accept their conclusion on the origin of the lectotype of *A. agama*. As a consequence, we regard *Agama agama* as the valid name for the widespread species of the complex in Western African and treat *Agama picticauda* as a junior synonym of *Agama agama*.

## Data Availability

All analyses are implemented using the code available in the R package *spider* v.1.3 (Brown *et al*.^38^).
